# Genetics of Type 2 Diabetes—Pitfalls and Possibilities

**DOI:** 10.3390/genes6010087

**Published:** 2015-03-12

**Authors:** Rashmi B. Prasad, Leif Groop

**Affiliations:** 1 Department of Clinical Sciences, Diabetes and Endocrinology, Lund University Diabetes Centre, Lund University, CRC, Skåne University Hospital SUS, SE-205 02 Malmö, Sweden; 2 Finnish Institute of Molecular Medicine (FIMM), Helsinki University, Helsinki 00014, Finland

**Keywords:** type 2 diabetes, genetics, heritability, genome-wide association studies, common variants, rare variants, parent-of-origin, systems biology

## Abstract

Type 2 diabetes (T2D) is a complex disease that is caused by a complex interplay between genetic, epigenetic and environmental factors. While the major environmental factors, diet and activity level, are well known, identification of the genetic factors has been a challenge. However, recent years have seen an explosion of genetic variants in risk and protection of T2D due to the technical development that has allowed genome-wide association studies and next-generation sequencing. Today, more than 120 variants have been convincingly replicated for association with T2D and many more with diabetes-related traits. Still, these variants only explain a small proportion of the total heritability of T2D. In this review, we address the possibilities to elucidate the genetic landscape of T2D as well as discuss pitfalls with current strategies to identify the elusive unknown heritability including the possibility that our definition of diabetes and its subgroups is imprecise and thereby makes the identification of genetic causes difficult.

## 1. The Diabetes Epidemic

Diabetes refers to a group of metabolic diseases characterized by hyperglycemia resulting from defects in insulin secretion, insulin action, or both [1]. The chronic hyperglycemia of diabetes is associated with long-term damage, dysfunction, and failure of different organs, especially the eyes, kidneys, nerves, heart, and blood vessels. Diabetes is currently the fastest-growing epidemic and has been ascribed to a collision between genes and the environment. Worldwide prevalence figures estimate that there were 382 million people living with diabetes in 2013 and that by 2035 this number will have risen to 592 million [2]. India and China have the highest reported prevalences of diabetes with 65 and 98 million in 2013, respectively, More than 90% of these cases are considered as T2D. In Europe, ~8% of the population is affected by diabetes, 90% of which is accounted for by T2D, making T2D the fastest-increasing disease in Europe and worldwide [2,3].

The T2D epidemic can largely be ascribed to the worldwide increase in obesity during the last 30 years, for instance, more than 60% of individuals older than 15 in the UK and US are overweight (BMI > 25) [4]. This has been ascribed to a collision between genes and the environment. The social determinants of environmental factors tend to vary across populations and have changed rapidly over the last decades. A traditional high energy-burning lifestyle has been replaced by a Western sedentary lifestyle with little or no exercise and consumption of an energy-dense diet. Meanwhile, genetic factors evolve at a slower rate across generations, and tend to favor selection of “energy-saving thrifty genotypes,” which might have been beneficial for individuals living in times of unstable food supply by storing energy in times of surplus [5]. While this hypothesis provides an appealing explanation to the obesity and T2D epidemic, formal proof for this hypothesis is still lacking.

Advances in genomic technology have initiated a myriad of novel genetic discoveries including more than 2,000 common variants contributing to risk of complex disease. While we have learned a great deal, a new biology about pathogenesis of these variants can only explain only a small portion of the total heritability of diabetes, leaving much of the underlying mechanisms unknown. The current review will try to explore the pitfalls with current strategies and elucidate possibilities to use genetics to enhance our understanding of the disease.

## 2. The Diabetes Spectrum

Diabetes encompasses a range of heterogeneous metabolic disorders characterized by the inability of the body to assimilate glucose and maintain glucose homeostasis. Diabetes has been traditionally subdivided into type 2 diabetes (T2D) and type 1 diabetes (T1D). However, this is a gross oversimplification of a rather complex situation. The concept of diabetes has grown over the past decades to the understanding that several different overlapping contributions from genetics and environment can lead to manifestations of varying forms disease. Contrary to being dichotomously distinct disorders, T1D and T2D can be considered rather as the two ends of a diabetes spectrum, with the intermediates comprising of maturity-onset diabetes of the young (MODY), latent autoimmune diabetes in adults (LADA) and other subtypes.

**Type 1 diabetes (T1D)**, also known as juvenile diabetes or insulin-dependent diabetes, is a chronic condition which is due to autoimmune destruction of pancreatic beta cells and is characterized by (nearly) complete absence of insulin secretion, and presence of autoantibodies including glutamic acid decarboxylase (GAD) antibodies, leading to dependence on insulin injections. It is most often diagnosed in children, adolescents or young adults less than 35 years old. The incidence of T1D varies based upon geography, age, gender, and family history [2]. Only 10% to 15% of newly diagnosed patients have a positive family history of T1D [6]. However, increased susceptibility to T1D can be inherited, because the average prevalence risk is 0.4% for children with no family history whereas ~6% when either parent has T1D, >30% when both parents are affected. There is a great difference in recurrence risk between dizygotic (8%) and monozygotic (50%) twins (30% risk within 10 years of diagnosis of the first twin) [7,8]. Interestingly, the risk in offspring of an affected mother is 2%–4%, whereas the risk of an affected father is as high as 5%–8% [9,10]. The sibling relative risk of T1D is estimated at 15 [7,11,12]. T1D results from interplay between genetic, epigenetic and environmental factors [13]. Genetic studies have been able to explain 80% of the heritability of T1D [14]. The main susceptibility genes currently accepted for T1D are the *HLA* class II alleles, which account for up to 50% of genetic T1D risk and non-*HLA* loci including the insulin gene, *CTLA4*, *PTPN22*, interleukin 2 receptor a (*IL2RA*), and others [15]. Environmental factors suggested so far include enterovirus infections including viruses of the picoRNA family, as they are seen more often among newly diagnosed T1D individuals than in the general population, and they precede the appearance of autoantibodies, environmental pollutants, gut flora variations and vitamin D exposure [16,17,18].

**LADA (Latent Autoimmune Diabetes in Adults)** is a common subgroup of diabetes accounting for about 7% of all diabetic patients in Europe. LADA is usually defined as GAD antibody-positive diabetes with onset greater than 35 years of age and no insulin requirement during the first 6 months after diagnosis [19,20,21]. The cutoff for defining GAD ab positivity is the same as for T1D. Therefore, LADA with high ab titers are found to the left of the spectrum close to T1D, whereas LADA with lower titers are to the right of the spectrum close to T2D [22]. A family history of any form of diabetes is a strong risk factor for the development of LADA [23].

**MODY (Maturity-Onset-Diabetes of the Young)** refers to monogenic forms of diabetes with well-defined mutations in more than 10 different genes, and this number is still increasing. The disease is characterized by autosomal dominant transmission of early-onset (<25 years) diabetes and varying degree of beta-cell dysfunction [24]. It was long debated whether the MODY genes would harbor common less-penetrant variants increasing risk of T2D; now, this seems to be the case for most of them including *HNF1A*, *HNF4A*, *HNF1B*, *GCK*, and *PDX1* [25,26,27]. MODY shows extreme allelic heterogeneity meaning that most MODY mutations are unique; to date, there are more than 200 mutations described in the *GCK* (MODY2) and *HNF1A* (MODY3) genes [28,29]. The appropriate diagnosis of MODY requires sequencing. With the advent of next-generation sequencing technologies, accurate MODY diagnoses are much more feasible today.

**Maternally inherited diabetes and deafness (MIDD)** is due to the A3242G mutation in mitochondrial DNA (mtDNA) [28,30]. As mtDNA is only transmitted from the mother, MIDD shows maternal transmission. In addition to hearing loss, many patients also display neurological problems similar to patients with the MELAS syndrome (mitochondrial myopathy, encephalopathy, lactic acidosis, and stroke), which is also caused by the same mutation in mtDNA.

**Neonatal diabetes** is defined as diabetes with onset at birth or during the first 6 months of life with both transient and permanent forms [28]. Mutations in several genes have been shown to cause neonatal diabetes (*KCJN11*, *SUR1*, *GCK*, *INS*, *etc.*), and an appropriate genetic diagnosis is a prerequisite for optimal treatment. Patients with mutations in the *KCJN11* gene do not only have severe diabetes but also developmental defects (DEND). These conditions are improved after switching from insulin treatment to treatment with large doses of sulfonylureas [31].

**Gestational Diabetes mellitus (GDM)** is a transitory form of diabetes which manifests as hyperglycemia during pregnancy which is clearly not overt diabetes and resolves itself post-partum. An estimated sibling risk ratio of 1.75 was reported for GDM [32,33]; however, changes in the diagnostic criteria have complicated retrospective identification of GDM cases and correct estimation of heritability. It has been observed that many of the GDM-associated variants overlap with T2D risk variants [34,35,36] which may partly explain the increased risk for T2D in women with previous GDM [37,38]. Despite being a transient condition, women with GDM are at increased risk for adverse pregnancy outcomes and fetal hyperinsulinism, and infants with macrosomia [39,40].

**Type 2 diabetes** is the most prevailing form, constituting 80%–90% of all reported diabetes cases. T2D is the result of a complex interplay between genetic, epigenetic and environmental factors. T2D develops when pancreatic beta cells can no longer produce enough insulin to compensate for the insulin resistance imposed by increasing obesity. There is no formal definition of T2D; patients who do not fulfill criteria of T1D, LADA, secondary diabetes (see below), or monogenic forms of diabetes are considered to have T2D. T2D is more often associated with increased age, wherein age of onset is usually over 35 years [14]. However, it is increasingly reported in adolescents in high risk countries such as India AND China [3]. Heritability of T2D is discussed below.

Finally, diabetes can develop secondary to pancreatic disease or other endocrine disorders and referred to as **secondary diabetes**.

**Figure 1:**
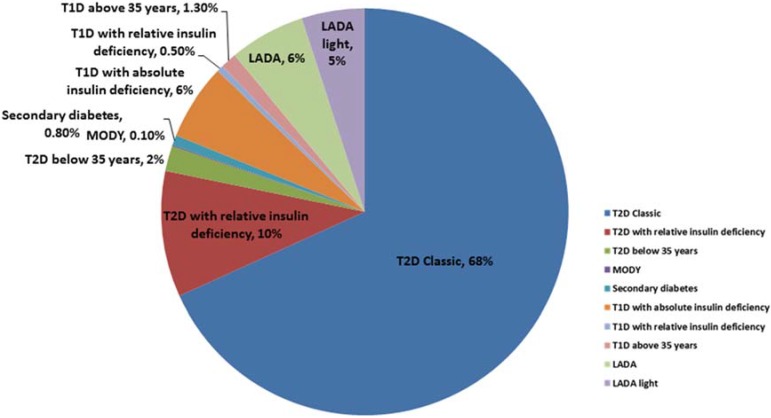
The spectrum of diabetes subgroups. The data is from the ANDIS project (All New Diabetics in Scania) (http://andis.ludc.med.lu.se) in April 2012 which at that time included 5,800 newly diagnosed diabetic patients aged 0–100 years. The criteria used for diagnosis are as follows: T1D: age at onset <35 years, C-peptide < 0.2nmol/L and GAD antibodies >20; T1D with relative insulin deficiency if C-peptide 0.2–0.6 nmol/L. T2D: age at onset >35 years, C-peptide > 0.6 nmol/L, GAD antibodies <10; T2D with relative insulin deficiency C-peptide 0.2–0.6 nmol/L. LADA (latent autoimmune diabetes in adults): age at onset > 5 years, GAD antibodies >20; LADA light if GAD antibodies 10–20. The data clearly illustrates the difficulty classifying diabetic patients at diagnosis with 19% unclassifiable.

All these forms of diabetes represent a range of genetic etiologies from the monogenic MODY variants to T2D, which is a complex heterogeneous polygenic disease with a strong environmental component. The ANDIS (All New Diabetics in Scania) project in southern Sweden represents a new attempt to reclassify diabetes into subgroups based upon genetic markers and biomarkers (Figure 1). The aim of ANDIS is to register all new cases of diabetes in Scania and improve diagnosis and treatment strategies. At the time of registration, blood samples are drawn to determine the presence of GAD antibodies, measure C-peptide, biobanking and for genetic analysis. The genetic data is used to classify the disease into subtypes and to study genetic causes of diabetes, diabetic complications and other disorders related to diabetes (http://andis.ludc.med.lu.se/all-new-diabetics-in-scania-andis/). A similar project has been initiated in Uppsala with the same goal (ANDIU—All new diabetics in Uppsala). The ANDIU study design is similar to the ANDIS study (http://www.andiu.se/english/).

## 3. Heritability of T2D

T2D clusters in families and it is well established that the risk of developing T2D depends on both genetic and environmental factors. However, heritability estimates have varied between 25%–80% in different studies; the highest estimates are seen in those studies with the longest follow-up periods. The lifetime risk of developing T2D is 40% for individuals who have one parent with T2D and almost 70% if both parents are affected [41]. Furthermore, the concordance rate of T2D in monozygotic twins is about 70%, while the concordance in dizygotic twins is only 20%–30%. The proband-wise concordance rates (number of affected twins having a diabetic co-twin) for monozygotic twins vary between 34% and 100% [42,43,44,45].

The relative risk for first degree relatives, *i.e.*, the risk of developing T2D if you have an affected parent or sibling compared to the general population, is approximately 3, and ~6 if both parents are affected [46]. However, these figures vary depending on the cohort and population studied.

The prevalence of T2D varies widely among populations, from a few percent among Caucasians in Europe to as high as 50% among Pima Indians in Arizona [47]. While part of the observed ethnic variability could be attributed to environmental and cultural factors, some of the variation seems to depend on genetic differences.

In spite of these reservations, there is no doubt the risk of T2D is partly determined by genetic factors, many of which have already been identified, and while each identified variant explains only a very small proportion of the risk of T2D in the human population, they have contributed to our understanding of disease pathogenesis. One should also keep in mind that the variance explained by a risk allele in a population is not necessarily an indicator of its importance in specific patients, nor is it proportional to the affected pathway’s importance or potential as a therapeutic target.

## 4. Possibilities

### 4.1. Search for the Genetic Basis of T2D

#### 4.1.1. Linkage Studies

The *CAPN10* gene on chromosome 10 encoding calpain 10, a cysteine protease with largely unknown functions in glucose metabolism, was the first T2D susceptibility gene to be identified through linkage studies [48]. Unfortunately, this locus has been difficult to replicate in subsequent studies. The greatest success in linkage studies relates to the discovery of variants in the *TCF7L2* as being associated with T2D. The DeCode team observed a rather modest linkage at a 10.5 Mb region on chromosome 10q, but decided to pursue and fine-map it thereby identifying the variant until the date showing the strongest association with T2D, an intronic variant (rs7903146) in the *TCF7L2* gene contributing to, but not fully explaining, the original linkage [49,50,51]. This association has since been confirmed in African, Asian and European populations rendering it the most consistently replicated genetic association with T2D to date, conferring a relative risk of ~1.4 [52].

#### 4.1.2. Candidate Genes for T2D

The first candidate gene reproducibly associated with T2D was *PPARG*, encoding the nuclear receptor PPAR-γ [53]. The PPAR-γ receptor is a molecular target for thiazolidinediones, a class of insulin-sensitizing drugs used to treat T2D, making it a very compelling candidate gene. The transcript expressed in adipose tissue has an extra exon B and a substitution of a proline for alanine at position 12 of this protein, which is seen in about 15% of the European population. This variant has been shown to be associated with increased transcriptional activity, increased insulin sensitivity and protection against T2D [53].

T2D risk variants in *KCJN11* were also discovered through candidate association studies [54,55]. *KCNJ11* codes for four subunits of the ATP-sensitive potassium (K-ATP) channel, the other four coded by another gene (*ABCC8*). The E23K polymorphisms in *KCNJ11* and P12A in *PPARG* putatively acted in an additive manner to increase T2D risk [56]. In pancreatic beta cells, ATP−potassium channels are crucial for the regulation of glucose-stimulated insulin secretion and are the target for the sulfonylureas which are oral hypoglycemic agents widely used in the treatment of T2D and for diazoxide, a potassium channel opener. Activating mutations in this gene also caused neonatal diabetes. Additionally, loss-of-function mutations in *KCNJ11* and *ABCC8* caused hyperinsulinemia in infancy [57].

#### 4.1.3. Genome Wide Association Studies (GWAS)

The development of new genotyping technologies and the realization that we inherit stretches of the genome together as haplotypes facilitated the cataloging of common variants (HAPMAP, 1000 genomes) and allowed for new possibilities to apply unbiased global approaches to screen millions of common variants for association with complex diseases. Several GWAS for diabetes were published in 2007, coined “Breakthrough of the Year” by *Science* magazine. The first was a GWAS on early-onset T2D reporting two new diabetes loci: *HHEX* and *SLC30A8* [58]. The ultimate proof of the value of GWAS for T2D came from three GWAS published back to back in *Science* in 2007; for the first time in the genetics of T2D, three different studies reported the same top findings [59,60,61]!

The first wave of GWAS was followed by a second wave combining existing or new GWAS into meta-analyses of >50,000 individuals [25]. A prerequisite for this was that many research groups could work together in consortia like DIAGRAM (DIAbetes Genetics Replication and Meta-analysis Consortium) and MAGIC (Meta-Analyses of Glucose-and Insulin-related traits Consortium). GWAS do not inevitably lead to identification of a gene or genes in a given locus associated with disease. Since the most strongly associated single nucleotide polymorphisms (SNPs) are often only markers for the functional variant responsible for the observed genetic effect and most associated regions harbor several genes, additional fine mapping of the loci in even larger sample sets is often necessary. To do this cost efficiently, a custom-designed chip, the so-called CardioMetabochip (Illumina) was been developed for metabolic/cardiovascular gene mapping. This chip contains ~200,000 polymorphisms selected to cover association signals from a wide range of metabolic disorders (T2D, lipid disorders, obesity and cardiovascular disease), and was designed to perform both deep replication of major disease signals and fine mapping of established loci. Meta-analysis of previous GWAS by the DIAGRAM consortium with an additional 22,669 T2D cases and 58,119 controls genotyped using the CardioMetabochip has recently added another eight new loci associated with T2D in the European population and 2 novel loci not previously reported in populations of European descent [62].

Recently, a number of GWAS and meta-analysis studies have also been performed in non-European cohorts, adding several new loci to the list of genome-wide significant associations [63,64,65,66,67,68,69,70]. Interestingly, it seems like most associations found in one ethnic group also show some evidence of association in populations with other ethnicities. Association of the same variants in more than one population adds to the robustness of the association. *KCNQ1*, for instance, was reported simultaneously as a T2D risk allele in two studies based on East Asian populations and further replicated in the European population as well [71,72]. Similarly, variants in *UBE2E2*, *C2CD4A-C2CD4B*, *ANK1*, *GRK5*, *RASGRP1*, *PAX4* and others were discovered in the East Asian population and replicated in Caucasians with variants in *PPAR*γ, *KCNJ11*, *TCF2*, *TCF7L2*, *CDKAL1*, *CDKN2A-CDKN2B*, *IDE-KIF11-HHEX*, *IGF2BP2*, whereas variants in *MTNR1B*, *SLC30A8*, *KCNQ1*, *CDC123*, *GLIS3*, *HNF1B*, *DUSP9* and others were identified in Caucasian populations and were also replicated in East Asian populations [63,64,65,66,67,68,69,70,73]. In addition, there are also some population specific risk variants whose association with T2D risk in other populations is yet to be ascertained. For instance, variants in *GLIS3*, *PEPD*, *ITM2-R3HDML-HNF4A*, *KCNK16*, *MAEA*, *GCC1-PAX4*, *PSMD6*, and *ZFAND* were discovered in populations of East Asian descent, *SLC16A11* in the Mexican population, and *TMEM163* in chr 2 in a North Indian population [63,74,75,76].

Trans-ethnic GWAS have been highly effective in the discovery of complex trait loci, and provide valuable insights into the genetic architecture of T2D across populations of diverse ancestry [70]. In addition, they enable fine-mapping by taking advantage of differences in genomic linkage disequilibrium across ethnically diverse populations, and facilitate prioritization of candidate genes [77]. However, allele frequencies can vastly vary between diverse populations and in terms of effects, and need to be taken into consideration while performing such meta-analyses. A method which takes into consideration the expected similarity in allelic effects between the most closely related populations, while allowing for heterogeneity between more diverse ethnic groups has been proposed to allow for this [78].

**Figure 2:**
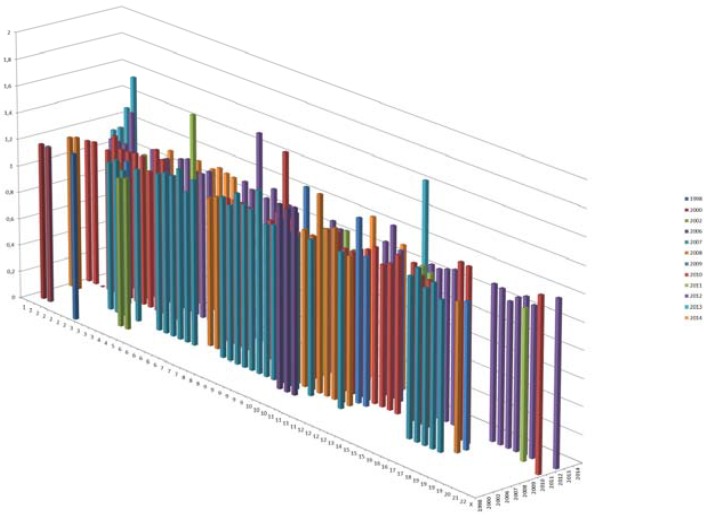
T2D risk variants. X axis shows the chromosomal location, Y shows the effect sizes and Z axis shows the year of discovery. Only 1 risk variant was reported in 1998; there were 2 in 2002, and today, we have a total of ~153 T2D variants.

**Figure 3:**
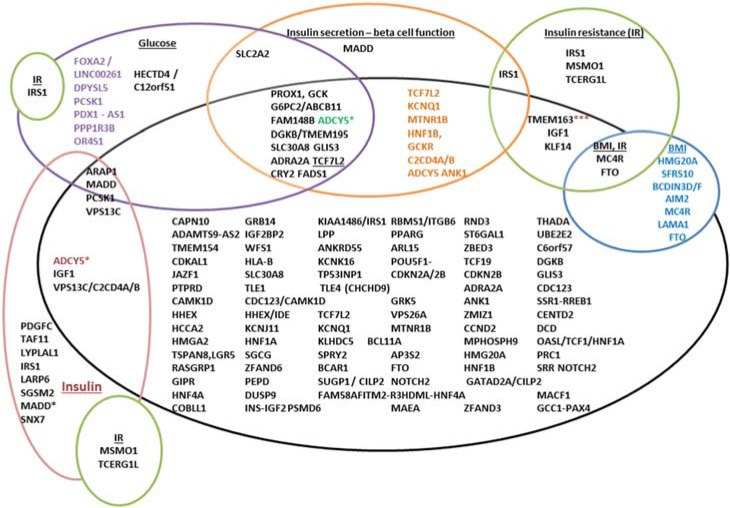
T2D and glycemic trait associated variants. The variants are represented by gene names here, which could indicate that the location is present either in the gene, or in the vicinity of the gene. The black circle represents T2D, and the gene names in black in this represent variants only associated with T2D. The overlapping circles indicate additional reporting associations for that variant for instance, *TCF7L2*, *KCNQ1*, *MTNR1B* *etc.*, are associated with T2D and also with beta-cell dysfunction. An *ADCY5* variant is associated with 2 h insulin adjusted for 2 h glucose; 2 h glucose/T2D (in brown) *** variants in TMEM163 are also associated with fasting insulin, *TCF7L2*—associated with fasting and 2 h glucose and *MADD* variants associated with fasting proinsulin, fasting glucose and HOMA-B.

In total, GWAS have provided ~153 variants for T2D mapping to >120 loci (Table 1, Figure 2 and Figure 3) as well as numerous loci for glucose or insulin-related traits (Table 2) and more are likely to come.

**Table 1:** Genetic loci associated with risk of T2D.

N	T2D Risk SNP	*Gene/Nearest Gene*	Gene Location	Chr	RA	OA	OR	TRAIT	Refs.
**1**	rs17106184	*FAF1*	intron	1	G	A	1.10	T2D	[70]
**2**	rs2296172	*MACF1*	coding - missense	1	G	A	1.10	2D	[79]
**3**	rs10923931	*NOTCH2*	intron	1	T	G	1.13	T2D	[4,80]
**4**	rs340874	*PROX1*	intergenic	1	C	T	1.07	Fasting glucose/HOMA B/T2D	[81]
**5**	rs243021	*BCL11A*	intergenic	2	A	G	1.08	T2D	[25]
**6**	rs243088	*BCL11A*	intergenic	2	T	A	1.07	T2D	[62]
**7**	rs2975760	*CAPN10*	intron	2	C	T	1.17	T2D	[48,82]
**8**	rs3792267	*CAPN10*	intron	2	G	A	1.17	T2D	[48,82]
**9**	rs7607980	*COBLL1*	coding-missense	2	T	C	1.14	T2D	[79]
**10**	rs560887	*G6PC2/ABCB11*	intron	2	T	C	1.03	Fasting glucose/T2D/HOMA B	[81]
**11**	rs780094	*GCKR*	intron	2	C	T	1.06	T2D/Fasting glucose/beta-cell function/triglycerides/fasting insulin	[81]
**12**	rs3923113	*GRB14*	intergenic	2	A	C	1.07	T2D	[62,65]
**13**	rs13389219	*GRB14*	intergenic	2	C	T	1.07	T2D	[62]
**14**	rs2943641	*IRS1*	intergenic	2	C	T	1.19	Fasting glucose/T2D/HOMAB, HOMA IR/AUC ins/AUC ratio/ISI	[83]
**15**	rs7578326	*KIAA1486/IRS1*	intron of uncharacterized LOC646736	2	A	G	1.11	T2D	[25]
**16**	rs7593730	*RBMS1/ITGB6*	intronic	2	C	T	1.11	T2D	[84]
**17**	rs7560163	*RND3*	intergenic	2	G	C	1.33	T2D	[67]
**18**	rs7578597	*THADA*	coding-missense	2	T	C	1.15	T2D	[4,80]
**19**	rs10200833	*THADA*	intron	2	G	C	1.06	T2D	[80,85]
**20**	rs6723108	*TMEM163*	intergenic	2	T	G	1.31	Decreased fasting plasma insulin/HOMA-IR/T2D	[74]
**21**	rs998451	*TMEM163*	intron	2	G	A	1.56	Decreased fasting plasma insulin/HOMA-IR/T2D	[74]
**22**	rs4607103	*ADAMTS9-AS2*	intron	3	C	T	1.09	T2D	[4,80]
**23**	rs6795735	*ADAMTS9-AS2*	intron	3	C	T	1.09	T2D	[4,80]
**24**	rs11708067	*ADCY5*	intron	3	A	G	1.12	T2D/2hr glucose/HOMA B	[81,86]
**25**	rs2877716	*ADCY5*	intron	3	C	T	1.12	2 h insulin adjusted for 2 h glucose/2 h glucose/T2D	[81,86]
**26**	rs11071657	*FAM148B*	intergenic	3	A	G	1.03	Fasting glucose/T2D/HOMA B	[81]
**27**	rs4402960	*IGF2BP2*	intron	3	T	G	1.11	T2D	[59]
**28**	rs1470579	*IGF2BP2*	intron	3	C	A	1.15	T2D	[26,59,72,87]
**29**	rs6808574	*LPP*	intergenic	3	C	T	1.07	T2D	[70]
**30**	rs1801282	*PPARG*	coding-missense	3	C	G	1.09	T2D	[59]
**31**	rs13081389	*PPARG*	intergenic	3	A	G	1.24	T2D	[25,53,59,87]
**32**	rs17036160	*PPARG*	intron	3	C	T	1.11	T2D	[85]
**33**	rs1797912	*PPARG*	intron	3	A	C	1.06	T2D	[85]
**34**	rs831571	*PSMD6*	intergenic	3	C	T	1.09	T2D	[63]
**35**	rs7647305	*SFRS10*	intergenic	3	C	T	1.08	BMI/obesity T2D	[88]
**36**	rs16861329	*ST6GAL1*	intron	3	G	A	1.09	T2D	[65]
**37**	rs6780569	*UBE2E2*	intergenic	3	G	A	1.21	T2D	[73]
**38**	rs6815464	*MAEA*	intron	4	C	G	1.13	T2D	[63]
**39**	rs7656416	*MAEA*	intron	4	C	T	1.15	T2D	[63,64]
**40**	rs6813195	*TMEM154*	intergenic	4	C	T	1.08	T2D	[70]
**41**	rs10010131	*WFS1*	intron	4	G	A	1.14	T2D	[4,89]
**42**	rs4689388	*WFS1*	nearGene-5	4	T	C	1.16	T2D	[83]
**43**	rs6446482	*WFS1*	intron	4	G	C	1.11	T2D	[25,89,90]
**44**	rs1801214	*WFS1*	coding-missense	4	T	C	1.13	T2D	[25,89,90]
**45**	rs459193	*ANKRD55*	intergenic	5	G	A	1.08	T2D	[62]
**46**	rs702634	*ARL15*	intron	5	A	G	1.06	T2D	[70]
**47**	rs4457053	*ZBED3*	intron of ZBED3-AS1	5	G	A	1.08	T2D	[25]
**48**	rs1048886	*C6orf57*	coding-missense	6	G	A	1.54	T2D	[91]
**49**	rs7754840	*CDKAL1*	intron	6	C	G	1.17	T2D	[25,27,59,60,66,73,92,93]
**50**	rs7756992	*CDKAL1*	intron	6	G	A	1.20	T2D	[27]
**51**	rs2206734	*CDKAL1*	intron	6	T	C	1.20	T2D	[25,27,59,60,66,73,92,93]
**52**	rs4712523	*CDKAL1*	intron	6	G	A	1.27	T2D	[25,27,59,60,66,73,83,92,93]
**53**	rs10946398	*CDKAL1*	intron	6	C	A	1.12	T2D	[25,27,59,60,66,73,92,93]
**54**	rs7766070	*CDKAL1*	intron	6	A	C	1.23	T2D	[25,27,59,60,66,73,92,93]
**55**	rs2244020 (rs9266650)	*HLA-B*	intergenic	6	G	A	1.09	T2D	[94]
**56**	rs1535500	*KCNK16*	coding-missense	6	T	G	1.08	T2D	[63]
**57**	rs3130501	*POU5F1-TCF19*	nearGene-5	6	G	A	1.07	T2D	[70]
**58**	rs9505118	*SSR1-RREB1*	intron	6	A	G	1.06	T2D	[70]
**59**	rs9470794	*ZFAND3*	intron	6	C	T	1.12	T2D	[63]
**60**	rs17168486	*DGKB*	intergenic	7	T	C	1.15	T2D	[62]
**61**	rs2191349	*DGKB/TMEM195*	intergenic	7	T	G	1.06	Fasting glucose, Homa B/T2D	[81]
**62**	rs6467136	*GCC1-PAX4*	intergenic	7	G	A	1.11	T2D	[63]
**63**	rs4607517	*GCK*	intergenic	7	A	G	1.07	Fasting glucose/T2D/HOMA B	[81]
**64**	rs864745	*JAZF1*	intron	7	T	C	1.10	T2D	[4,80]
**65**	rs849134	*JAZF1*	intron	7	A	G	1.13	T2D	[25,80]
**66**	rs12113122	*JAZF1*	intron	7	G	C	1.55	T2D	[85]
**67**	rs972283	*KLF14*	intergenic	7	G	A	1.07	Reduced insulin sensitivity T2D	[25]
**68**	rs516946	*ANK1*	intron	8	C	T	1.09	T2D	[62]
**69**	rs515071	*ANK1*	intron	8	G	A	1.18	T2D Reduced beta-cell function	[62,64]
**70**	rs13266634	*SLC30A8*	coding-missense	8	C	T	1.19	T2D	[58]
**71**	rs11558471	*SLC30A8*	UTR-3	8	A	G	1.15	Fasting glucose, HOMA B T2D	[25,58,59,81,87,92,93]
**72**	rs3802177	*SLC30A8*	UTR-3	8	G	A	1.26	T2D	[25,58,59,81,87,92,93]
**73**	rs896854	*TP53INP1*	intron	8	T	C	1.06	T2D	[25]
**74**	rs10965250	*CDKN2A/2B*	intergenic	9	G	A	1.20	T2D	[25,27,59,60,66,73,92,93]
**75**	rs2383208	*CDKN2A/2B*	intergenic	9	A	G	1.19	T2D	[25,27,59,60,66,73,92,93]
**76**	rs7018475	*CDKN2A/2B*	intergenic	9	G	T	1.35	T2D	[25,27,59,60,66,73,92,93]
**77**	rs564398	*CDKN2A/2B*	intergenic	9	T	C	1.12	T2D	[25,27,59,60,66,73,92,93]
**78**	rs10757282	*CDKN2A/2B*	intergenic	9	C	T	1.14	T2D	[25,27,59,60,66,73,92,93]
**79**	rs10811661	*CDKN2B*	intergenic	9	T	C	1.20	T2D	[25,59,60,66,68,80,87,92]
**80**	rs7034200	*GLIS3*	intron	9	A	C	1.03	Fasting glucose/T2D/HOMA B	[81]
**81**	rs7041847	*GLIS3*	intron	9	A	G	1.10	T2D	[63,66]
**82**	rs10814916	*GLIS3*	intron	9	C	A	1.11	T2D	[63,66,81]
**83**	rs17584499	*PTPRD*	intron	9	T	C	1.57	T2D	[95]
**84**	rs2796441	*TLE1*	intergenic	9	G	A	1.07	T2D	[62]
**85**	rs13292136	*TLE4 (CHCHD9)*	intergenic	9	C	T	1.11	T2D	[25]
**86**	rs553668	*ADRA2A*	UTR-3	10	A	G	1.42	T2D	[96]
**87**	rs10885122	*ADRA2A*	intergenic	10	G	T	1.04	Fasting glucose/HOMA B/T2D	[81]
**88**	rs12779790	*CDC123, CAMK1D*	intergenic	10	G	A	1.11	T2D	[4,80]
**89**	rs11257655	*CDC123/CAMK1D*	intergenic	10	C	T	1.15	T2D	[66,69,80]
**90**	rs10906115	*CDC123/CAMK1D*	intergenic	10	A	G	1.13	T2D	[66,69,80]
**91**	rs10886471	*GRK5*	intron	10	C	T	1.12	T2D	[66]
**92**	rs5015480	*HHEX*	intergenic	10	C	T	1.13	T2D	[25,58,59,92,93]
**93**	rs1111875	*HHEX/IDE*	intergenic	10	C	T	1.13	T2D	[59]
**94**	rs7903146	*TCF7L2*	intronic/promoter	10	T	C	1.35	T2D, fasting glucose,.2 h glucose	[51]
**95**	rs4506565	*TCF7L2*	intron	10	T	A	1.34	Fasting glucose, HOMA B T2D	[25,27,51,58,59,60,61,80,87,92,93,97,98,99]
**96**	rs7901695	*TCF7L2*	intron	10	C	T	1.37	T2D	[25,27,51,58,59,60,61,80,87,92,93,97,98,99]
**97**	rs1802295	*VPS26A*	UTR-3	10	A	G	1.08	T2D	[65]
**98**	rs12571751	*ZMIZ1*	intron	10	A	G	1.08	T2D	[62]
**99**	rs11603334	*ARAP1*	UTR-5	11	G	A	1.13	T2D fasting proinsulin levels/fasting glucose/	[100]
**100**	rs1552224	*CENTD2*	intergenic	11	A	C	1.14	T2D	[25]
**101**	rs11605924	*CRY2*	intron	11	A	C	1.04	Fasting glucose/HOMA B/T2D	[81]
**102**	rs174550	*FADS1*	intron	11	T	C	1.04	Fasting glucose/T2D/HOMA B	[81]
**103**	rs2334499	*HCCA2*	intergenic	11	T	C	1.35	T2D	[101]
**104**	rs3842770	*INS-IGF2*	intron	11	A	G	1.18	T2D - African American	[94]
**105**	rs5219	*KCNJ11*	coding-missense	11	T	C	1.14	T2D	[54,59,60,87,99]
**106**	rs5215	*KCNJ11*	coding-missense	11	C	T	1.14	T2D	[54,59,60,87,99]
**107**	rs2237895	*KCNQ1*	intron	11	C	T	1.45	T2D	[71]
**108**	rs231362	*KCNQ1*	intron	11	G	A	1.08	T2D	[25]
**109**	rs163184	*KCNQ1*	intron	11	G	T	1.22	T2D	[62,71]
**110**	rs2237892	*KCNQ1*	intron	11	C	T	1.25	Reduced beta-cell function T2D	[25,71,72,92,95]
**111**	rs10501320	*MADD*	intron	11	G	C	1.01	T2D fasting proinsulin levels/fasting glucose	[100]
**112**	rs10830963	*MTNR1B*	intron	11	G	C	1.09	T2D	[102]
**113**	rs1387153	*MTNR1B*	intergenic	11	T	C	1.09	Reduced beta-cell function T2D	[25,95,102]
**114**	rs7138803	*BCDIN3D/FAIM2*	intergenic	12	A	G	1.11	BMI/obesity T2D	[88,103]
**115**	rs11063069	*CCND2*	intergenic	12	G	A	1.12	T2D	[62]
**116**	rs1153188	*DCD*	intergenic	12	A	T	1.08	T2D	[80]
**117**	rs1531343	*HMGA2*	intron of pseudogene	12	C	G	1.10	T2D	[25]
**118**	rs9668162	*HMGA2*	intron	12	G	C	1.26	T2D	[85]
**119**	rs7305618	*HNF1A*	intergenic	12	C	T	1.14	T2D	[25,68]
**120**	rs35767	*IGF1*	nearGene-5	12	G	A	1.04	Fasting insulin/T2D/HOMA IR	[81]
**121**	rs10842994	*KLHDC5*	intergenic	12	C	T	1.10	T2D	[62]
**122**	rs4275659	*MPHOSPH9*	intron	12	C	T	1.06	T2D	[70]
**123**	rs7957197	*OASL/TCF1/HNF1A*	intron of OASL	12	T	A	1.07	T2D	[25]
**124**	rs7961581	*TSPAN8, LGR5*	intergenic	12	C	T	1.09	T2D	[4,80]
**125**	rs9552911	*SGCG*	intron	13	G	A	1.63	T2D	[104]
**126**	rs1359790	*SPRY2*	intergenic	13	G	A	1.15	T2D	[69]
**127**	rs2028299	*AP3S2*	UTR-3	15	C	A	1.10	T2D	[65]
**128**	rs7172432	*C2CD4A/B*	intergenic	15	A	G	1.14	Reduced beta-cell function, T2D	[73]
**129**	rs7178572	*HMG20A*	intergenic	15	A	G	1.09	lean T2D	[65,93]
**130**	rs7177055	*HMG20A*	intergenic	15	A	G	1.08	T2D	[62]
**131**	rs8042680	*PRC1*	intron	15	A	C	1.07	T2D	[25]
**132**	rs7403531	*RASGRP1*	intron	15	T	C	1.10	T2D	[66]
**133**	rs4502156	*VPS13C/C2CD4A/B*	intergenic	15	T	C	1.07	fasting proinsulin levels T2D	[100]
**134**	rs11634397	*ZFAND6*	intergenic	15	G	A	1.06	T2D	[25]
**135**	rs7202877	*BCAR1*	intergenic	16	T	G	1.12	T2D	[62]
**136**	rs8050136	*FTO*	intron	16	A	C	1.17	Increased BMI, reduced insulin sensitivity, T2D	[25,60,61,80,87,93,99,105]
**137**	rs9939609	*FTO*	intron	16	A	T	1.25	T2D (obese)	[25,60,61,80,87,93,99,105]
**138**	rs11642841	*FTO*	intron	16	A	C	1.13	T2D	[25,60,61,80,87,93,99,105]
**139**	rs4430796	*HNF1B*	intron	17	G	A	1.19	Reduced beta-cell function T2D	[66,106,66,107,108]
**140**	rs7501939	*HNF1B*	intron	17	T	C	1.09	T2D	[106]
**141**	rs391300	*SRR*	intron	17	G	A	1.28	T2D	[95]
**142**	rs4523957	*SRR*	nearGene-5	17	T		1.27	T2D	[95]
**143**	rs8090011	*LAMA1*	intron	18	G	C	1.13	lean T2D	[93]
**144**	rs17782313	*MC4R*	intergenic	18	C	T	1.06	BMI/T2D	[88,103]
**145**	rs12970134	*MC4R*	intergenic	18	A	G	1.08	T2D/BMI/waist circumference/insulin resistance	[62,109]
**146**	rs3794991	*GATAD2A/CILP2*	intron, intergenic	19	T	C	1.12	T2D	[62,85]
**147**	rs8108269	*GIPR*	intergenic	19	G	T	1.05	T2D	[62]
**148**	rs3786897	*PEPD*	intron	19	A	G	1.10	T2D	[63]
**149**	rs10401969	*SUGP1/CILP2*	intron	19	C	T	1.13	T2D	[62,85]
**150**	rs6017317	*FITM2-R3HDML-HNF4A*	intergenic	20	G	T	1.09	T2D	[63]
**151**	rs4812829	*HNF4A*	intron	20	A	G	1.09	T2D	[65]
**152**	rs5945326	*DUSP9*	intergenic	X	A	G	1.27	T2D	[25]
**153**	rs12010175	*FAM58A*	intron	X	G	A	1.21	T2D	[66]

**Table 2:** Genetic loci associated with glycemic traits.

N	SNPs	*Gene/Nearest Gene*	Gene Location	Chr	Effect Allele	Other Allele	Effect	TRAIT	Refs.
**1**	rs9727115	*SNX7*	intron	1	G	A	0.0133	fasting proinsulin levels adjusted for fasting glucose	[100]
**2**	rs2785980	*LYPLAL1*	intergenic	1	T	C	0.017	fasting insulin	[110]
**3**	rs4675095	*IRS1*	intron	2	A	T	−0.006/−002	fasting glucose/HOMA-IR	[81]
**4**	rs2943634	*IRS1*	intergenic	2	C	A	0.025	fasting insulin, CAD	[110]
**5**	rs1371614	*DPYSL5*	intron	2	T	C	0.022	Fasting glucose	[110]
**6**	rs11920090	*SLC2A2*	intron	3	T	A	0.02	Fasting glucose/HOMA B/HBA1C	[81]
**7**	rs17046216	*MSMO1*	intron	4	A	T	0.18; 0.19	Fasting Insulin; insulin resistance	[111]
**8**	rs4691380	*PDGFC*	intron	4	C	T	0.021	fasting insulin	[110]
**9**	rs6235	*PCSK1*	coding-missense	5	G	C	0.0394/−0.014	fasting proinsulin levels/Fasting glucose	[100]
**10**	rs13179048	*PCSK1*	intergenic	5	C	A	0.018	Fasting glucose	[110]
**11**	rs4646949	*TAF11*	nearGene-3	6	T	G	0.020	fasting insulin	[110]
**12**	rs6943153	*GRB10*	intron	7	C	T	0.0154	FG, FI	[26]
**13**	rs4841132	*PPP1R3B*	intergenic	8	A	G	0.030	Fasting glucose	[110]
**14**	rs7077836	*TCERG1L*	intergenic	10	T	C	0.28; 0.34	Fasting Insulin ; insulin resistance	[111]
**15**	rs7944584	*MADD*	intron	11	A	T	0.021	Fasting proinsulin/Fasting glucose/Homa B	[81]
**16**	rs10838687	*MADD*	intron	11	T	G	0.0253	fasting proinsulin levels	[100]
**17**	rs1483121	*OR4S1*	intergenic	11	G	A	0.015	Fasting glucose	[110]
**18**	rs2074356	*HECTD4/C12orf51*	intron	12				1-h plasma glucose	[112]
**19**	rs2293941	*PDX1 - AS1*	intron	13	A	G	0.016	Fasting glucose	[110]
**20**	rs17271305	*VPS13C*	intron	15	G	A	0.07	2hr glucose/2-h insulin, adjusted for 2-h glucose	[86]
**21**	rs1549318	*LARP6*	intergenic	15	T	C	0.0192	fasting proinsulin levels	[100]
**22**	rs4790333	*SGSM2*	intron	17	T	C	0.0154	fasting proinsulin levels	[100]
**23**	rs10423928	*GIPR*	intron	19	A	T		2 h glucose/Insulinogenic index/AUCins/gluc/2-h insulin, adjusted for 2 h glucose/T2D	[86]
**24**	rs6048205	*FOXA2/LINC00261*	intergenic/nearGene-5	20	A	G	0.029	Fasting glucose	[110]

#### 4.1.4. Rare Variants

Rare variants are more recent and therefore more likely to be those that arose lately in an extended pedigree. Of course, natural selection removes the more deleterious variants before they reach a high frequency; risk alleles for diseases should thus be enriched at lower frequencies. The idea that there are unique rare variant combinations in families and their role in disease etiology is referred to as “clan genomics” [113]. Current data suggests that the combined effects of rare and common variants contribute in varying degrees to disease causation, even linkage; in fact, rare alleles may explain the majority of heritability [114]. Next-generation sequencing provides even denser coverage of genetic variation rendering detection of causal rare variants more feasible. Whole genome sequencing (WGS) of 2,630 Icelanders and imputation into 11,114 Icelandic cases and 267,140 controls followed by testing in Danish and Iranian samples revealed variants in *PAM* and *PDX1* as risk of T2D [115] (Table 3). Array-based genotyping and exome sequencing on a small founder population from Greenland revealed a nonsense p.Arg684Ter variant (allele frequency of 17%) in *TBC1D4* associated with higher concentrations of 2 h glucose and serum insulin [116]. Whole-exome sequencing in a Latino population revealed a rare missense variant in HNF1A (c.1522G > A [p.E508K]) was associated with type 2 diabetes prevalence [117]. Additionally, rare variants associated with glycemic traits were discovered through exome sequencing of 9,717 individuals from the METSIM study, Finland [118] (Table 3).

**Table 3:** Rare risk and protective loci associated with T2D and glycemic traits.

N	SNPs	*GENE/Nearest Gene*	Gene Location	Chr	Refs.
**1**	rs35658696	*PAM*	coding-missense	5	[118]
**2**	rs78408340	*PAM*	coding-missense	5	[118]
**3**	rs36046591	*PPIP5K2*	coding-missense	5	[118]
**4**	p.Lys34Serfs*50	*SLC30A8*	coding-missense	8	[119]
**5**	p.Arg138*	*SLC30A8*	coding-missense	8	[119]
**6**	rs3824420	*KANK1*	coding-missense	9	[118]
**7**	rs505922	*ABO*	Intronic	9	[118]
**8**	rs60980157	*GPSM1*	coding-missense	9	[118]
**9**	p.Leu5Val (20)	*ATG13*	coding-missense	11	[118]
**10**	p.Ile131Val (1)	*ATG13*	coding-missense	11	[118]
**11**	p.Gln249Pro (3)	*ATG13*	coding-missense	11	[118]
**12**	p.Arg392Trp (1)	*ATG13*	coding-missense	11	[118]
**13**	p.Leu427Gln (3)	*ATG13*	coding-missense	11	[118]
**14**	p.Gly434Arg (488)	*ATG13*	coding-missense	11	[118]
**15**	p.X406Gly (200)	*ATG13*	coding-missense	11	[118]
**16**	rs35233100	*MADD*	coding-missense	11	[118]
**17**	p.Arg279Cys (324)	*TBC1D30*	coding-missense	12	[118]
**18**	p.Pro746Leu (427)	*TBC1D30*	coding-missense	12	[118]
**19**	c.1522G > A [p.E508K]	*HNF1A*	coding-missense	12	[117]
**20**	rs76895963	*CCND2*	intergenic	12	[115]
**21**	rs75615236	*CCND2*	intergenic	12	[115]
**22**	rs150781447	*TBC1D30*	coding-missense	12	[118]
**23**	rs2650000	*HNF1A*	Intergenic	12	[118]
**24**	Chr. 13: g.27396636delT	*PDX1*	coding-missense	13	[119]
**25**	p.Tyr416Cys (78)	*SGSM2*	coding-missense	17	[118]
**26**	p.Thr789Pro (3)	*SGSM2*	coding-missense	17	[118]
**27**	p.Val996Ile (236)	*SGSM2*	coding-missense	17	[118]
**28**	rs61741902	*SGSM2*	coding-missense	17	[118]

#### 4.1.5. Structural Variants

Most studies to date have been limited to SNP leaving structural polymorphisms relatively unexplored. However, since common structural variants are likely to be tagged by surrounding SNPs, they are unlikely to explain a large proportion of missing heritability. A recent study identified a common copy number variation (CNV), CNVR5583.1 (*TSPAN8*), as associated with T2D [120]. This association could be convincingly replicated by previously typed SNPs that tag the CNV [80].

#### 4.1.6. Protective Variants

The average T2D risk variant frequency is 54% in the general population, which raises the question: Is T2D the default condition? If so, then does carrying protective variants makes a difference in the disease susceptibility? Studies have been performed to address this question but included controls that, despite a clustering of risk factors for T2D, have escaped the disease. A rare (0.66%) loss of function mutation (R138X) was detected in the *SLC30A8* gene in the Botnia region from Finland and subsequently replicated in a massive effort applying the Exome chip to >150,000 individuals from other European countries. Also, the DeCode group had identified another LoF mutation, a frameshift mutation which also was enriched in the non-diabetic Icelandic population. The SLC30A8 gene encodes the islet zinc transporter 8 with a putative effect on insulin secretion. Notably, a common variant in the same gene increases susceptibility to T2D, whereas autoantibodies to T1D predispose to T1D.

Collectively, carriers of these protein-truncating mutations have a 65% lower risk of T2D [119]. Other studies based on Icelandic, Danish and Iranian populations identified a low frequency variant in *CCND2* that reduced T2D risk by half [115]. Moreover, variants in TCF2 were found to be protective against T2D [106]. It is also likely that the more recent variants that are only a few generations old segregate in families and could be detected though sequencing in families.

#### 4.1.7. The Genetic Architecture of T2D

Genetic architecture of a complex phenotype is defined by the number, frequencies and effect sizes of causal alleles. Many hypotheses have been proposed to define the genetic architecture of T2D: one hypothesis suggested that the unexplained heritability lies in a large number of common variants with low additive effects and that the disease represents the extremes of a normal distribution [121]. Another proposed that rare alleles might be responsible for effects observed with common variants (synthetic associations) and explain a majority of the heritability [113,122,123]. One argument against common and more so against rare variants is that they would have been removed from the population by natural selection [47]; however, this is not a valid argument for a disease like T2D whereby the penetrance of the genetic effect depends strongly on interactions with the environment, especially since this environment has changed in recent years and the genetic risk variants could have been neutral or even beneficial before the introduction of the Westernized lifestyle.

While extreme models are excluded by the present data based on epidemiological, linkage and GWAS, models wherein rare variants explain a little (<25%) or a lot (>80%) of heritability remain consistent [124]. Large next-generation sequencing studies in families will hopefully answer the questions about the role of rare variants in complex diseases. There is already substantial evidence for parent-of-origin effects on T2D risk, and studies are ongoing to explore this further. Structural polymorphisms and microRNAs add a further layer of complexity and have not yet been exhaustively studied.

The genetic architecture could also be influenced by gene-gene interactions (epistasis) where rare, variants with high penetrance, could act jointly with common alleles to increase risk of disease. The extent of allelic heterogeneity seems to be less pronounced for the common form of T2D than for monogenic forms like MODY. Moreover, there could be differences in the genetic predisposition to T2D due to phenotypic heterogeneity within T2D cases. For instance, lean T2D cases are likely to carry a disproportionately high load of type 2 diabetes risk alleles [93].

### 4.2. Functions of Associated Genes

Most identified diabetes loci have not been mechanistically tied to the disease. While loci are commonly referred to by the names of genes located close to them, only a few are close to strong biological candidates, e.g., the melatonin receptor (*MTNR1B*) and the insulin receptor substrate-1 (*IRS1*). For others, like *TCF7L2* and *GIPR*, the evidence is quite strong that an intronic SNP is the causal SNP. Melatonin receptor 1B (*MTNR1B*) has been associated with both fasting glucose and T2D risk [102,125,126]. Melatonin works as a chronobiotic factor, adjusting the timing of the biological clock. Its receptors are present in the pancreas and melatonin is proposed to contribute to the nocturnal lowering of insulin in humans. The *MTNR1B* risk genotype is associated with impaired early insulin release to both oral and intravenous glucose and insulin secretion deteriorates over time in the risk allele carriers [125]. The proposed mechanism by which *MTNR1B* polymorphism could predispose to T2D involves altered expression of *MTNR1B* in pancreatic β-cells leading to decreased cAMP/cGMP concentrations via G proteins and, thereby, impaired insulin secretion.

The insulin receptor substrate 1 (*IRS1*) gene encodes a protein that mediates insulin’s control of various cellular processes by transmitting signals from the insulin receptor to intracellular signaling pathways. The C allele of rs2943641 has been shown to be associated with insulin resistance and increased risk of diabetes. The genetic variant causes reduced basal levels of IRS1 protein and decreased insulin induction of IRS1-associated phosphatidylinositol-3-hydroxykinase activity in human skeletal muscle biopsies [83].

*TCF7L2* is a transcription factor playing an important role in the Wnt signaling pathway. The risk allele is associated with decreased insulinogenic index and lower disposition index, suggesting a reduced capacity for insulin secretion in relation to insulin sensitivity. Since it was identified as a diabetes gene, it has been shown to be important for several vital functions in the pancreatic islet, including pancreas development, determination of beta-cell mass, maintenance of the secretory function of mature beta cells, and regulation of insulin production and processing [127,128].

The incretin hormone *GIP* (glucose-dependent insulintropic polypeptide) promotes pancreatic β-cell function by potentiating insulin secretion and β-cell proliferation. The *GIP* receptor (*GIPR*) locus showed association to postprandial insulin levels in a meta-analysis performed by the MAGIC consortium but was surprisingly not associated with risk of diabetes in the DIAGRAM+ study [25,86]. The reason seems to be that the same variant results in decreased BMI, which neutralizes the effect of the SNP on risk of T2D. GIP influences expression of the inflammatory cytokine *OPN* in islets, which in turn has protective effects on β-cell proliferation and potentially apoptosis [129].

Many of the other identified loci can be sub-grouped based on their association with other phenotypes with a key role in T2D etiology. Exploration of the effects of T2D-associated variants on glucose and insulin traits in non-diabetic populations has shown that most of the known loci act through an effect on insulin secretion rather than insulin resistance (Table 1) [25,130,131,132].

Fasting glucose-raising alleles of the *MADD*, *GIPR*, *GCK*, *FADS*, *DGKB*, *PROX1*, *TCF7L2*, *SLC30A8* and *C2CD4B* loci have all been associated with either abnormal insulin processing or secretion, whereas *GCKR* and *IGF1* are associated with OGTT-based disposition indices and β-cell function [131]. The DIAGRAM+ consortium observed that three loci (*TCF7L2*, *ARAP1* and *CDKAL1*) were associated with reduced fasting insulin also suggestive of β-cell dysfunction, whereas the T2D risk alleles at *PPARG*, *FTO*, *IRS1* and *KLF14* were associated with higher fasting insulin, indicating a primary effect on insulin action [25].

#### Translational Studies

The *ADRA2A* (adrenergic receptor alpha 2) locus was recently identified as a T2D risk locus after first having been positionally mapped in congenic GK rats where it was associated with impaired insulin granule docking and reduced β-cell exocytosis [130]. Human carriers of the *ADRA2A* risk variant (rs553668) have reduced fasting insulin and decreased insulin secretion as a consequence of increased expression of the *ADRA2A* receptor in pancreatic islets. It is well known that epinephrine excess can suppress insulin secretion and cause diabetes. The α_2A_AR antagonist yohimbine enhances insulin release *in vitro* in islets from organ donors carrying the risk allele to levels similar to those in non-risk carriers. A randomized clinical study was performed blocking α_2A_AR pharmacologically to increase insulin secretion T2D patients with the rs553668 risk allele. Yohimbine administration enhanced 30 min insulin, corrected insulin response and disposition index in the risk group, making secretion similar to patients carrying the low-risk allele. Insulin secretion defect in patients carrying the *ADRA2A* risk genotype could be corrected by α2AAR antagonism [133]. This demonstrated the potential application of genetic risk variants to guide therapeutic interventions that target the underlying pathophysiology: one step closer to individualized medicine.

## 5. Pitfalls

### 5.1. Heritability Estimates

Heritability parameters facilitate understanding the genetic architecture of complex traits such as T2D. However, the >80 loci identified explain less than 20% of the heritability of T2D. There are many possible explanations for the missing heritability, including assumptions made about the genetic architecture of the disease and the definitions of heritability. The estimations of heritability explained assumes that only additive affects determine disease risk and that the risk follows the liability threshold model, *i.e.*, that the genetic and environmental effects sum up to form a normal distribution of liability and that disease arises in individuals surpassing a certain threshold in the distribution [134]. If these assumptions are not true, the estimate of heritability explained will not be correct.

Intrauterine effects can also affect heritability estimates because monozygotic twins are often monochorionic which results in growth retardation compared to dizygotic twins, and low birth weight is associated with increased risk of T2D later in life. Furthermore, there could be other explanations to the “missing heritability” problem. However, one should keep in mind that heritability can only be estimated from the most recent generations for which information on affection status is available, whereas most of the variants studied thus far are ancestral variants hundreds of generations old. We do not know whether these ancestral variants (that have modest effects and have escaped purifying selection) can really explain the diabetes epidemic we see in the most recent generations or whether this can be ascribed to rare variants with stronger effects.

Moreover, heritability estimates are based on “top” SNPs from GWAS associations; novel methods have been proposed which take into account the (i) scale 0–1 scale as opposed to liability (2) ascertainment bias and (3) quality control of the GWAS SNPs. Estimating the proportion of variance explained by all SNPs in GWAS as opposed to only the most significantly associated SNPs could result in a more detailed estimate of heritability [135]. Applying an approach that considers all SNPs on the chip could in fact explain a much larger proportion of the “narrow-sense” heritability (>50%) supporting the existence of numerous yet unidentified loci with smaller effects [25,136,137].

### 5.2. Parent of Origin Effects

The risk of T2D in offspring is greater if the mother has T2D compared to if the father is affected in contrast to T1D, where the risk of T1D in offspring is greater if the father is affected [138,139]. Sex-specific parental effects have been reported for insulin response to the oral glucose load, with male offspring of diabetic mothers showing the lowest insulin values, as well as influencing HDL concentrations [138]. One potential explanation for this could be preferential parental specific transmissions of risk alleles to offspring, which is often associated with DNA methylation and imprinting. Epigenetic modifications have the potential to be stable and heritable across cell divisions and [140,141] manifest as parent-of-origin effects. A large-scale family-based study on the Icelandic population determined that variants in *KCNQ1* and *KLF14* show stronger effects on T2D when the risk allele is transmitted from the mother than from the father [101,142] and was replicated in later studies [143] including our own.

The conflict hypothesis suggests that imprinting arose due to a genomic tug-of-war between mothers and fathers over the use of maternal resources in the fetus. The paternal imprinting maximizes the utilization of intrauterine resources to the offspring which would increase his evolutionary fitness whereas the maternal imprinting tries to minimize this in order to conserve it for her future offspring [144]. Conversely, the co-adaptation hypothesis suggests that imprinted genes coevolve to optimize parental care of offspring. While there is insufficient evidence to support either theory, the significant role of imprinting in defining paternal and maternal effects has nevertheless been consistently established [145].

The intrauterine environment plays a significant role in determining fetal programming. It has been shown that poor nutrition can affect fetal growth, can produce permanent changes in glucose-insulin metabolism, and often results in low birth weight [146] This can induce permanent changes in metabolism and affect chronic disease susceptibility as proposed by the DoHAD hypothesis [147]. If this intrauterine programming results in a reduced β-cell mass, it could predispose to diabetes later in life when the insulin requirements increase as a consequence of obesity resulting in insulin resistance. *KCNQ1* could represent an example of fetal programming wherein the maternally expressed gene was mono-allelically expressed in fetal tissues and bi-allelically expressed in adult tissues [148].

Dissection of genetic parent-of-origin effects requires genotype data from families and only heterozygous parents are informative yielding reduced power for relatively rare variants. However, long-range phasing and imputation methods allow for predicting genotypes with a great likelihood, thus making this a valuable method to find “surrogate” parents even if DNA exists from only a few family members. When the paternal and maternal allele have effects in opposite directions—as, for instance in a situation where the maternal allele could confer risk while the paternal allele could be protective—such an association would be almost impossible to detect in a traditional case-control GWAS. However, some novel POE detection methods allow detection of imprinting effects from differences in the phenotypic variance of heterozygotes in very large case-control studies [149]. Parent-of-origin effects could explain a large portion of the missing heritability and must be taken into consideration in investigations of genetic T2D susceptibility.

### 5.3. Gene−Gene and Gene−Environment Interactions

Gene−gene interactions, or epistasis, have been suggested as a possible explanation for difficulties in replicating genetic association in complex diseases [150]. The standard statistical methods used in association studies are usually limited to analysis of single marker effects and thereby do not account for interactions between markers. Previous attempts to study epistasis in complex diseases have focused on interactions between candidate regions [151,152]. However, the recent abundance of GWAS data has made a comprehensive search across the genome more feasible. Some studies have attempted to account for epistasis in GWAS using a two-step approach in which significant SNPs are tested against each other or against all other SNPs in the study with variable results [153,154]. The main problem when studying epistasis is power, since interaction between loci with modest effects is difficult to detect without extremely large sample sizes. However, some studies have pointed at novel tests to increase power [155]. Thorough studies in diabetes addressing epistasis using this approach are missing. Furthermore, a recent paper by Eric Lander and co-workers provided compelling evidence that gene−gene interaction can also contribute to missing heritability by causing “phantom heritability” that inflates the estimated narrow sense heritability of the trait [156].

Gene−environment interactions are equally difficult to study but are likely to play an important role in T2D development. The epidemic of T2D only dates back 50 years, and it is quite obvious that during this period that only the environment, and not the genes, have changed. However, the genetic architecture determines our response to the environment. Genetic variants could affect specific metabolic processes to make an individual more susceptible to the harmful effects of a poor diet but also personality traits that make an individual more or less likely to over-consume and live a sedentary lifestyle. It will however be a formidable task to identify the environmental triggers for most of the genetic variants increasing susceptibility to diabetes as this will require very large studies with precise information on diet, exercise, energy expenditure, *etc.*

### 5.4. Epigenetics

The environment can also influence the expression of the genome, and ultimately the phenotype, via the epigenome. Even though the DNA sequence is not changed, the phenotype is altered by epigenetic modifications of gene expression by mechanisms including methylation of DNA, posttranslational modification of histones, or activation of microRNAs. Changes to the phenotype can be at the level of the cell, tissue, or whole organism.

It is tempting to speculate that environmental factors such as diet and exercise can change the level of DNA methylation and thereby cause changes in gene expression, but evidence that DNA methylation contributes to the increase in T2D is still lacking. Epigenetic mechanisms may however play a role in progression of the disease by inducing glucotoxicity in islets and predispose to diabetic complications [157]. Elevated glucose is a prerequisite to this condition and it is well established that cells can memorize changes in glucose concentrations. For example, two large studies, the UKPDS and DCCT studies, showed that an initial good metabolic control was associated with reduced frequency of diabetic complications decades later. The advanced “metabolic memory” hypothesis suggests that this is because glucose can induce histone modifications in endothelial cells that can be remembered long after [158].

### 5.5. Non-Coding RNAs−microRNAs

Non-coding RNAs have recently emerged as important regulators of gene expression and function. MicroRNAs (miRNAs) naturally regulate programs of gene expression. Altered miRNA function has been shown to contribute to human disease, and manipulation of specific miRNAs is now being explored as a novel therapeutic modalities [159]. The efficiency of miRNAs binding to target transcripts depends on both the sequence and the intra-molecular structure of the transcript. SNPs can contribute to alterations in the structure of regions flanking them, thereby influencing the accessibility for miRNA binding. Several studies have implicated miRNAs in diabetes and inflammation and common SNPs change the target sequence of miRNAs in several T2D susceptibility loci (http://miracle.igib.res.in/dbSMR) [160,161]. Other forms of non-coding RNAs, such as piRNAs (PIWI-interacting RNAs), snoRNAs (small nucleolar RNAs), lincRNAs (long intergenic non-coding RNAs), and lncRNAs (long non-coding RNAs), might also contribute to the development of diabetes. For example, the *CDKN2A*/*B* region on chromosome 9 is associated with T2D, as well as cardiovascular disease and a number of other disorders. This region harbors an lncRNA, ANRIL (non-protein coding *CDKN2B-AS1 CDKN2B* antisense RNA 1), which can potentially modify and explain some of these associations [162].

### 5.6. The T1D-T2D Paradox

T1D and T2D can be considered two extremes of the diabetes spectrum and share a few similarities in manifestation of underlying physiology including hyperglycemia, insulin deficiency and development of complications. However, the genetics of T1D and T2D vastly differ, with very few T2D susceptibility loci showing an association with T1D. Notable exceptions include the *PPARG* Pro12Ala variant, *MTNR1B*, *HNF1A*, *GLIS3*, 6q22.32 and novel loci near the MHC, which harbor the *HLA* class II genes associated with about half the T1D risk [70,163,164,165]. Based on these studies, the mechanisms underlying T1D and T2D appear to be intrinsically distinct. The distribution of T2D risk SNPs should be more random in a T1D patient; however, this does not seem to be the case. The strongest T2D−SNP in the TCF7L2 gene almost seems to protect from T1D. T1D risk variants for *BCAR1*, *GLIS3* and *RAD51L1* were protective for T2D whereas for those in C6orf173, *COBL* and C10orf59, the effects were coincident [62]. Also, it has been reported that *APOC3* haplotypes increase risk of T1D; however, the same variants increase risk of T2D in lean carriers while having a protective effect in overweight carriers [166]. Common variants in *SLC30A8* are associated with an increased risk of T2D, and rare variants with a protective effect [58,119]. Puzzlingly, *SLC30A8* was also found to be a major autoantigen eliciting 60%–80% autoantibodies in new-onset T1D patients [167].

Curiously, gene variants associated with T1D underwent recent positive selection and have been increasing in prevalence. There is more selection in alleles increasing, rather than decreasing, susceptibility to T1D. This is indicative of an evolutionary benefit, wherein these variants were possibly protective against viruses and bacterial infections. However, no such link has been reported for type 2 diabetes risk variants, as could be expected from the thrifty genotype hypothesis [168]. In terms of genetics, T2D seems to have more in common with that of cancer rather than T1D [169]. It has been suggested there may rather be a specific yin−yang relationship between cancer and T2D, with too much cell proliferation resulting in cancer and insufficient proliferation of pancreatic islets resulting in T2D [169].

LADA (latent autoimmune diabetes in adults) is considered an intermediary form between T1D and T2D, also referred to as type 1.5 diabetes. There is much less information available as to the genetic basis of LADA compared to T1D and T2D. One way to understand this would be to assess to what extent LADA shares genetic similarities with T1D and T2D. The HLA locus, conferring 50% of the genetic susceptibility of T1D also shows similar associations with LADA, with a few differences. The T1D variant *PTPN22* shows a weak association with LADA. Data on the *INS* class I variable number tandem repeats (VNTRs) has been inconclusive and associations have been reported with both T1D and T2D wherein the short tandem repeat is associated with T1D and the long with T2D. While previously several T1D-associated variants could be tested for association with LADA, it was not until the discovery of the common variant in the *TCF7L2* gene being strongly associated with T2D that the genetic contribution of T2D to LADA could really be tested. This variant is clearly associated with LADA and T2D but not with T1D. This indicates that LADA is indeed a genetic admixture of T1D and T2D.

There are more lessons to be learned from differences between T1D and T2D rather than similarities. Elucidating the genetic heterogeneity of the spectrum of diabetes disorders will help us in understanding the mechanisms underlying the phenotypic heterogeneity of diabetes and would be a step towards individualized therapy.

### 5.7. Systems Biology

A restricted focus on the genome through GWAS provides limited insights into the molecular mechanisms driving disease and is akin to a snapshot of the genetics of the disease. To obtain an understanding of disease pathogenesis, it is important to analyze the GWAS data in the context of complementary follow-up analyses including DNA methylation and histone modifications, expression profiling under conditions relevant for the disease, related protein analysis, and analysis of genotype−phenotype associations. Global transcriptome profiling in relevant tissues such as pancreas has facilitated identification and cataloging of a wide array of transcription-based events in the pathogenesis of T2D [170]. For example, by combining GWAS information with metabolomics, it has been possible to identify strong associations between SNPs and metabolic reactions that otherwise would have been missed [171]. Network or pathway-based approaches, including enrichment in pre-defined pathways by, for example, KEGG [172] (http://www.genome.jp) and Gene Ontology (GO) (http://www.geneontology.org), have also been used to identify disease genes for various diseases. Thus, an integrative approach of several data types is likely to discover disease genes that would not be identified by the use of classical GWAS approaches. This was also illustrated in a recent study of human islets identifying novel candidate genes for T2D based upon expression differences and co-expression with known T2D genes as well as protein−protein interaction analyses [173,174]. Integration of GWAS data with such data could therefore facilitate a systems-based understanding of the pathogenic mechanisms.

## 6. Conclusions

The technical revolution in the field of genetics has allowed identification of numerous genetic variants that associate with T2D. The genetic landscape of T2D susceptibility is as yet incomplete, thus far only explaining a small proportion of the total heritability of diabetes. Many possibilities to dissect the architecture of T2D etiology have emerged in the form of large-scale genetic studies, meta-analyses and sequencing in families. It has already greatly contributed to our understanding of disease mechanisms by identifying pathways that could not be linked to diabetes by existing hypothetical models, even though many genetic findings are very recent and have yet to make their contribution to our knowledge about diabetes pathogenesis. However, one must bear in mind that diabetes is probably a much more diverse disease than the current subdivision into T1D and T2D implies and more precise subdivision into subgroups may both facilitate the investigation of T2D genetics and pave the way for more individualized treatment. A holistic systems biology approach will also be required to obtain a complete picture of how genetic variation leads to diabetes. The rapid technology development during the past years holds promises that this will be possible in a not-too-distant future.
